# Delayed Appearance of High Altitude Retinal Hemorrhages

**DOI:** 10.1371/journal.pone.0011532

**Published:** 2011-02-17

**Authors:** Daniel Barthelmes, Martina M. Bosch, Tobias M. Merz, Benno L. Petrig, Frederic Truffer, Konrad E. Bloch, Timothy A. Holmes, Philippe Cattin, Urs Hefti, Miriam Sellner, Florian K. P. Sutter, Marco Maggiorini, Klara Landau

**Affiliations:** 1 Department of Ophthalmology, University Hospital Zurich, Zurich, Switzerland; 2 Department of Intensive Care Medicine, Inselspital, Bern University Hospital and University of Bern, Bern, Switzerland; 3 Institut de Recherche en Ophtalmologie, Sion, Switzerland; 4 Pulmonary Division, University Hospital Zurich, Zurich, Switzerland; 5 Center for Integrative Human Physiology, University of Zurich, Zurich, Switzerland; 6 Medical Image Analysis Center (MIAC), University of Basel Faculty of Medicine, Basel, Switzerland; 7 Department of Surgery, State Hospital Liestal, Liestal, Switzerland; 8 Medical Intensive Care Unit, University Hospital Zurich, Zurich, Switzerland; University of Las Palmas de Gran Canaria, Spain

## Abstract

**Background:**

Retinal hemorrhages have been described as a component of high altitude retinopathy (HAR) in association with altitude illness. In this prospective high altitude study, we aimed to gain new insights into the pathophysiology of HAR and explored whether HAR could be a valid early indicator of altitude illness.

**Methodology/Principal Findings:**

28 mountaineers were randomly assigned to two ascent profiles during a research expedition to Mt. Muztagh Ata (7546 m/24,751 ft). Digital fundus photographs were taken prior to expedition at 490 m (1,607 ft), during expedition at 4497 m (14,750 ft = base camp), 5533 m (18,148 ft), 6265 m (20,549 ft), 6865 m (22,517 ft) and 4.5 months thereafter at 490 m. Number, size and time of occurrence of hemorrhages were recorded. Oxygen saturation (SpO_2_) and hematocrit were also assessed. 79% of all climbers exhibited retinal hemorrhages during the expedition. Number and area of retinal bleeding increased moderately to medium altitudes (6265 m). Most retinal hemorrhages were detected after return to base camp from a high altitude. No post-expeditional ophthalmic sequelae were detected. Significant negative (SpO_2_ Beta: −0.4, p<0.001) and positive (hematocrit Beta: 0.2, p = 0.002, time at altitude Beta: 0.33, p = 0.003) correlations with hemorrhages were found.

**Conclusions/Significance:**

When closely examined, a very large amount of climbers exhibit retinal hemorrhages during exposure to high altitudes. The incidence of retinal hemorrhages may be greater than previously appreciated as a definite time lag was observed between highest altitude reached and development of retinal bleeding. Retinal hemorrhages should not be considered warning signs of impending severe altitude illness due to their delayed appearance.

## Introduction

Altitude-related illness, which includes acute mountain sickness (AMS), high altitude cerebral edema (HACE) and high altitude retinopathy (HAR)[1], [2], occurs in unacclimatized individuals exposed to hypobaric hypoxia at high altitudes.[3] Independent risk factors for the development of high altitude illness are the altitude reached, individual susceptibility and rate of ascent.[4], [5] Climbers affected by AMS suffer from a variety of nonspecific symptoms such as headache, nausea, insomnia, dizziness, lassitude or fatigue.[6] In some mountaineers symptoms can progress and develop into HACE, the end-stage of AMS, which is probably due to hypoxia-induced increase in cerebral blood flow coupled with decreased integrity of the blood-brain barrier and cytotoxic edema.[7] It is diagnosed by detection of ataxia, altered consciousness or both in a person with AMS. If not treated accordingly, this illness is lethal.

Many individuals who ascend to heights above 3000 m develop HAR, reported as engorgement and tortuosity of the retinal vessels, and optic disc hyperemia and swelling, retinal hemorrhages, nerve fiber layer infarction, and even vitreous hemorrhage.[8] The regulation of the cerebral circulation has been reported to behave similarly to that of the retinal vessels under hyper- and hypoxic conditions.[9], [10] Therefore common mechanisms may play a role in the pathophysiology of HAR, AMS and HACE. Whether HAR is potentially related to CNS dysfunction at high altitudes is still a matter of debate.[11], [12]

Clarke et al.[11] doubt that the appearance of isolated retinal hemorrhages is a warning sign of impending cerebral edema. In contradistinction, Wiedman and Tabin[12] indicated a possible association between HAR and HACE. This hypothesis is supported by recent studies showing that altered autoregulation of the cerebral blood flow[13] and vasogenic cerebral edema[14] may cause AMS and HACE.

The Muztagh Ata (7546 m/24,751 ft) medical research expedition study was performed by researchers from the medical specialties of pulmonology, neurology, hematology and ophthalmology. The overall aim of this prospective large scale study was to assess hypoxic changes of the body in a holistic approach. Our goal in this study was to gain new insights in the pathophysiology of HAR by obtaining frequent field fundus photographs and correlate potential ocular fundus abnormalities with concomitant ocular changes as well as high altitude-associated cerebral symptoms and signs. Finally, we explored whether HAR could be a valid early indicator of altitude illness or even HACE.

## Methods

### Study design and outcomes

**Figure 1:**
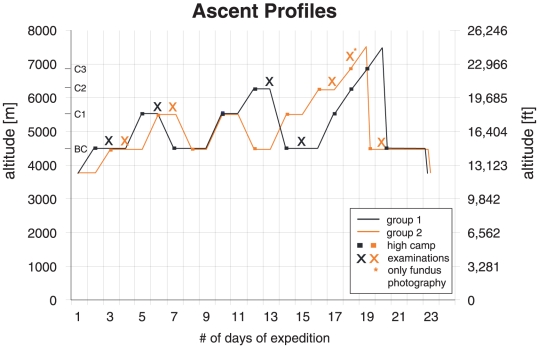
Ascent profile of both groups of climbers with indication of high camps and examination time points.

In this prospective, multidisciplinary study[5], [15], [16] 32 mountaineers were randomly assigned to two ascent profiles (Fig. 1) during an expedition to Mt. Muztagh Ata (7546 m/24,751 ft) in western China. Both ascent groups started the expedition at 3750 m/12,300 ft, then continued to base camp (BC1 = 4497 m/14,750 ft), camp 1 (C1 = 5533 m/18,148 ft), camp 2 (C2 = 6265 m/20,549 ft), camp 3 (C3 = 6865 m/22,517 ft) and to the summit (7546 m/24,751 ft) within 20 (group 1) and 19 (group 2) days. Average ascent rates were 190 and 200 m/d (623 and 656 ft/d), respectively (Fig. 1). Included were healthy, physically fit, experienced mountaineers of either gender and between 20 to 65 years of age without any ophthalmic pathology. They needed to have reached at least C2 and had to have available fundus photographs at second base camp (BC2) examination. Exclusion criteria were any type of ocular, cardiac or respiratory disease, or a history of high altitude pulmonary edema or high-altitude cerebral edema (HACE) after a rapid ascent (<3 nights) to altitudes below 3500 m. Time to every new examination camp (in days) was recorded from the start of the expedition above 3750 m/12,300 ft ( = time at altitude).

The study was approved by the Ethics Committee of the University Hospital in Zurich, and adheres to the tenets of the Declaration of Helsinki (1983 revision). All subjects gave written consent prior to the examinations after having been informed of the nature and goals of the research expedition.

General and ophthalmic examinations were performed 1 month prior to the expedition (ZH1) and 4.5 months after return (ZH2), at the University Hospital of Zurich (490 m/1607 ft).

Eye examinations included: digital fundus photography (in ZH: Zeiss FF 450 plus camera, Carl Zeiss AG, Oberkochen, Germany; on Mt Muztagh Ata: handheld fundus camera Genesis-D, Kowa Inc., Tokyo, Japan). At every examination, 12 photographs were taken of each eye according to a standardized protocol, where the macula and the entire periphery were documented in a clockwise manner at half-minute intervals. Number, size (in optic disc areas), distance from center of optic disc (in optic disc diameters) to center of hemorrhage, and altitude at which hemorrhages occurred were assessed by two independent ophthalmologists. The custom made program employed to measure size and distance of retinal hemorrhages was developed in MATLAB (R2007a, The MathWorks Inc., Natick MA; U.S.A.), and accounted for different magnification between the cameras and image distortion due to different angles while taking images of the fundus periphery. This enabled direct comparison of retinal hemorrhages independent of the employed fundus camera and zoom factor.

Ancillary ophthalmic examinations included: fluorescein angiography, vessel diameter measurements of the temporal superior branch retinal artery and vein (arbitrary units [AU]) as described earlier,[16] and optic disc evaluation.[15] Furthermore, perifoveal retinal blood flow measurements were performed utilizing the blue field simulation (BFS-1000, Oculix Inc., Berwyn, USA) technique[16] resulting in time-averaged velocity (BFS-Vel mm/s) of leukocytes. Subfoveal choroidal blood flow measurements were done with a portable confocal laser Doppler flowmetry[17] (LDF) system (Institut de Recherche en Ophtalmologie, Sion, Switzerland)[16] permitting localized blood flow measurements (LDF flow- derived from velocity and volume in AU).[18] Intraocular pressure measurements were performed with the portable Tono-Pen XL (Reichert Inc., Depew, NY, U.S.A.).[19] Visual fields before and after the climb were obtained with the dynamic strategy (G2) program of a full-projection perimeter (Octopus 101; Haag-Streit AG, Koeniz/Bern, Switzerland). Amsler grid self-evaluation of the central visual field was performed individually every second day during the climb and recorded in the personal diary.

#### Vital Parameters

Diastolic (P_A.dia_) and systolic (P_A.sys_) brachial artery blood pressure were measured with a sphygmomanometry device. Mean systemic blood pressure was calculated as follows: P_A.dia_ + (1/3)×(P_A.sys_ − P_A.dia_). Perfusion pressure of the eye resulted from subtracting intraocular pressure (IOP) from 2/3 of the mean systemic blood pressure.[20]

Hematocrit was measured in capillary blood samples and values were determined after centrifugation onsite (HAEMATOKRIT 2010, Hettich, Switzerland).

Daily oximetry was performed in the evening during rest in a standing position with a finger pulse oximeter (Onyx 9500 SportStat, Nonin Medical Inc., Plymouth, MN, U.S.A.). Stable values after at least 3 minutes were recorded.[5]

Cerebral acute mountain sickness (AMS-c) scores were assessed daily utilizing the Environmental Symptom Questionnaire (ESQ III).[15], [21] A score of ≥0.7 reliably identifies a person with AMS.[21] The AMS-c score reflects symptoms of altered cerebral function in conjunction with the experience of being ill. Diagnosis of HACE was made when headache and an AMS-c score of ≥0.7 were present either with change in mental status or with ataxia.

Drug intake was recorded. Intake of non steroidal anti-inflammatory agents was allowed by protocol, whereas other medication was taken only upon prescription by the independent expedition physician.

Except for fluorescein angiography and full-field perimetry, all climbers underwent all examinations mentioned above during the expedition, on the subsequent day upon arrival at each new camp (Fig. 1).

### Statistical analysis

Data are expressed as mean ± standard deviation (SD) if normally distributed and as median with interquartile ranges if distribution was shown not to be normal. Normality was determined using the Kolmogorov-Smirnov test. Differences between the groups were assessed using unpaired Student's t-test with Welch correction or the Mann Whitney U test, where appropriate. Fisher's exact test was used to analyze whether there was a statistically significant difference in the number of retinal hemorrhages and number of Roth spots between both groups. Two separate multiple regression analyses, including testing for collinearity were performed to analyze relations between the number and total area of hemorrhages and different predictors pertaining either to systemic changes or to changes in ocular circulation parameters. A two sided α-error (*P*-value) of less than 0.05 was prospectively defined as statistically significant. Statistical analysis was performed with the software Statistica 6 (StatSoft Inc., Tulsa, OK, U.S.A.) and SPSS 13.0 (SPSS Inc., Chicago, IL, USA).

#### Role of the funding source

Funding was utilized for device and further mountaineering material acquisition in addition to financing the great amount of logistics needed prior, during and after the expedition. The study sponsors did not play any role in design or implementation of the study.

## Results

Four participants out of the 32 initially included in the ophthalmology study were excluded because of incomplete data collection during the expedition resulting in 28 mountaineers available for analysis. Twenty-six climbers reached 6850 m and two (both from group 1) 6250 m as the maximum height during the expedition; 17 mountaineers (9 in group 1 (Gr1), 8 in group 2 (Gr2)) reached the summit. Due to bad weather conditions, Gr1 had to descend from C2 to BC instead of the designated further ascent to C3. Hence, BC2 examinations in Gr1 were performed after the descent from C2 and due to lack of manpower only group 2 (Gr2) could be documented at C3 and BC2 at the end of the expedition. There is no statistically significant difference between groups 1 (n = 13) and 2 (n = 15) concerning age ([mean±SD]: 45±12 and 44±8, respectively) and gender (female:male: 2∶11 and 4∶11, respectively).

**Figure 2:**
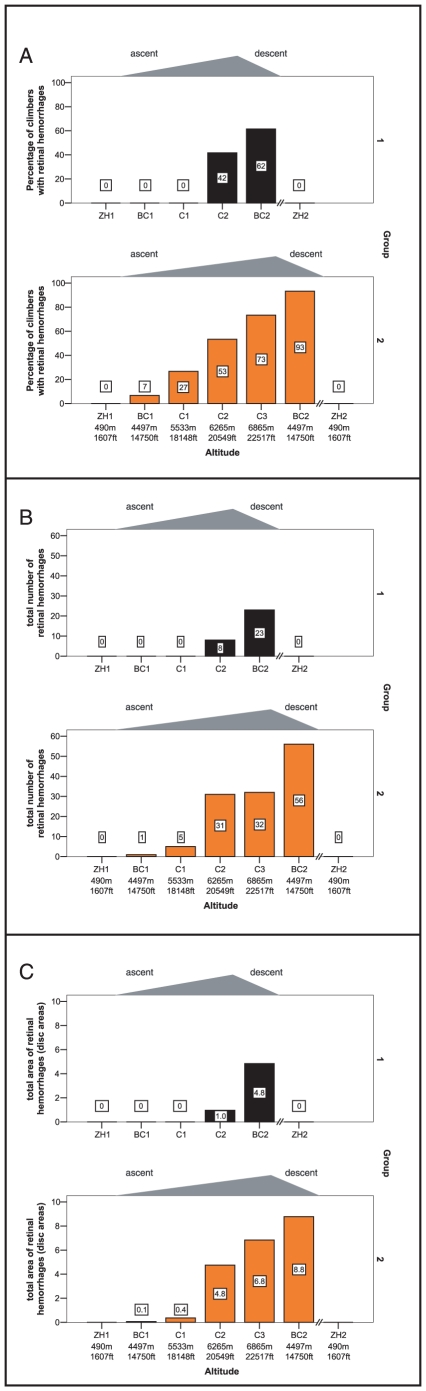
Graphs describing hemorrhages in both groups of climbers at different altitudes. Panel A: total number of hemorrhages; panel B: total area of hemorrhages; panel C: percentage of mountaineers with hemorrhages in at least 1 eye.

**Figure 3:**
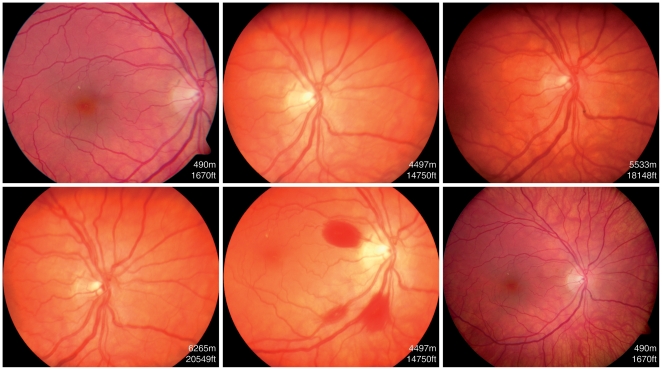
Fundus photographs of a climber from group 1 showing the development of retinal hemorrhages during the course of her climb. Note the white-centered hemorrhage localized at the temporal inferior branch of the retinal artery.

Of 28 mountaineers, 22 (79%) exhibited retinal hemorrhages in at least one eye during the expedition. Details and an example can be found in Table 1, Fig. 2(Panels A–C) and Fig. 3. An increase in both number and area of hemorrhages in both groups occurred during ascent to C2. In Gr2, although further ascending, the number of hemorrhages stagnated whereas the area of bleeding increased. In both groups the majority of hemorrhages (number and area) were detected after descent to base camp. The total number of hemorrhages increased by 288% (Gr1) and 175% (Gr2) upon descent to BC2, whereas the total bleeding area increased by 480% (Gr1) and 130% (Gr2). The mean distance from the center of the optic disc to the center of the hemorrhage was 1.5±0.8 disc diameters in all climbers; no statistically significant differences were detected between groups and altitudes. No hemorrhages were sighted within the fovea.

**Table 1:** Summary of total number, total area of retinal hemorrhages (in optic disc areas) and percentage of climbers with hemorrhages per group at different altitudes.

Altitude	Group	Number of climbers examined	Number of climbers with retinal hemorrhages	Total number of hemorrhages observed	Total area of hemorrhages observed (in disc areas)	Percentage of climbers with hemorrhages
ZH1	1	13	0	0	0.00	0
BC1	1	13	0	0	0.00	0
C1	1	13	0	0	0.00	0
C2	1	12	5	8	0.96	42
BC2	1	13	8	23	4.84	62
ZH2	1	13	0	0	0.00	0
ZH1	2	15	0	0	0.00	0
BC1	2	15	1	1	0.07	7
C1	2	15	4	5	0.37	27
C2	2	15	8	31	4.75	53
C3	2	15	11	32	6.83	73
BC2	2	15	14	56	8.77	93
ZH2	2	15	0	0	0.00	0

No hemorrhages could be detected either before or after the expedition. Roth spots were detected in 3 climbers in Gr1 and in 6 in Gr2 without a statistically significant difference between groups.

No changes in fluorescein angiography were found after the expedition (ZH2) compared to initial normal examinations (ZH1). Full-field perimetry results were unchanged at ZH2 compared to ZH1. Neither metamorphopsia nor central scotomas were recorded in Amsler grid tests. The only visual disturbances reported were floaters by one participant in whom retinal bleeding was so extensive that vitreous hemorrhage occurred. An overview of changes in the systemic parameters (AMS-c, SpO_2_, perfusion pressure and hematocrit) and drug intake is presented in Table 2.

**Table 2:** An overview of changes in the systemic parameters (AMS-c, SaO_2_ in %, mean arterial blood pressure in mmHg, perfusion pressure in mmHg and hematocrit in %) and drug intake.

	ZH1	BC1	C1	C2	C3	BC2	ZH2
AMS-c score	0 (0–0)	0 (0–0.88)	0.09 (0–5.6)	0.09 (0–2.42)	0.23 (0–1.38)	0 (0–0.18)	0 (0–0)
SpO_2_	98±0.84	84±2.56	75±6.33	73±6.04	65±3.49	87±4.87	98±0.75
Perfusion Pressure	45±7.44	45±6.6	48±6.21	49±4.94	not available	48±6.07	50±3.91
Hematocrit	not available	44±3.12	45±3.73	50±4.22	not available	not available	43±2.12
Diamox	0	0	0	0	0	3	0
Adalat	0	0	0	0	0	1	0
Steroids	0	0	0	0	0	2	0
Ibuprofen	0	12	19	14	7	8	0

There was no statistically significant difference in total area and number of hemorrhages between groups at the same altitudes. This allowed us to merge the data for further analysis.

Separate multiple regression analyses with total number of hemorrhages per altitude per climber and also total area of hemorrhages per altitude per climber as the dependent factors and environmental and systemic changes within each climber (SpO_2_, hematocrit, time at altitude, optic disc swelling, AMS-c score, age) yielded partial correlation coefficient (Beta) and significance level (p value) pairs noted in Table 3. Results of same analysis with independent predictors SpO_2_ and ocular circulation parameters (BFS-V, perfusion pressure, LDF-Flow, arterial diameter, venous diameter) are also shown in Table 3. Collinearity testing did not reveal significant results. Regression analysis of drug intake did not yield a significant partial correlation to total number (Beta = −0.009, p = 0.14) or total area of hemorrhages (beta = −0.08, p = 0.19) when corrected for age, time at altitude and AMS-c scores. Two climbers suffered from early HACE during the expedition. A plot of their number and total area of hemorrhages against those of the climbers without any signs of HACE did not show any systematic deviation or obvious differences (data not shown). The 9 climbers with Roth spots were also evaluated in comparison to all others. They showed more (at C1 p = 0.003, C2 p = 0.008, BC2 p<0.001) and larger areas of retinal bleeding (at C1 p = 0.025, C3 p = 0.015, BC2 p = 0.006) and had significantly lower oxygen saturation levels at C1. No statistically significant differences in hematocrit, AMS-c scores and age were found.

**Table 3:** Results of multiple regression analysis performed with total number and total area of hemorrhages and various independent predictors.

Independent predictors:	Total number of hemorrhages		Total area of hemorrhages	
	Beta	p-value	Beta	p-value
SpO2	−0.40	<0.001	−0.42	<0.001
Hematocrit	0.22	0.002	0.23	0.001
Time at Altitude	0.33	0.003	0.30	0.006
Optic disc swelling	0.13	0.08	0.17	0.019
AMS-c score	−0.09	0.15	−0.12	0.06
Age	−0.09	0.13	−0.1	0.13
SpO_2_	−0.38	<0.001	−0.40	<0.001
BFS-V	−0.13	0.07	−0.11	0.12
Perfusion Pressure	−0.05	0.42	−0.09	0.15
LDF-Flow	0.06	0.29	0.03	0.65
arterial diameter	−0.12	0.19	−0.06	0.49
venous diameter	0.01	0.91	0.03	0.73

## Discussion

A vast majority of climbers, namely 79%, exhibited retinal hemorrhages during the expedition of which most were detected only after descent from a high altitude. Our novel findings are important since they indicate that retinal hemorrhages cannot be considered as warning signs of severe altitude sickness. Furthermore, the data suggest that the incidence of retinal hemorrhages may have been underestimated in previous studies.

Our climbers with very low oxygen saturation showed more retinal bleeding throughout the expedition. Also, the higher the ascent and the longer the duration at high altitudes, the higher was the occurrence of retinal hemorrhages. This concurs with a study by McFadden et al. revealing that subjects who developed HAR during maximal exercise showed a lower SaO_2_ during exercise than those who did not present with HAR at any time at high altitude.[8] If the occurrence of retinal hemorrhages were an immediate effect of the ascent (i.e., increasing hypoxia), many more hemorrhages should have been detected during the ascent (e.g. in group 2 from C2 to C3) as opposed to the surprising increase in hemorrhages observed upon descent. Group 1 (Gr1) exhibited fewer hemorrhages than group 2 (Gr2), possibly due to the fact that Gr1 had final examinations at BC2 after a lower maximal altitude (C2) as compared to Gr2, in which the BC2 examinations were performed after the descent from C3. Our hypothesis stating a correlation between the degree of hypoxia and the number of hemorrhages is supported by the results of the statistical analyses, including our multiple regression analysis. The results strongly suggest a correlation between the degree of hypoxia - severity and time of exposure - and the number of hemorrhages: many more climbers in Gr2 (93%) - who had had sustained longer and more extensive systemic hypoxia- showed retinal hemorrhages at BC2 than in Gr1 (62%).

Independent of the amount of hypoxia, the time of occurrence of retinal bleeding is delayed in relation to the ascent for both groups (Fig. 2). Hypoxic damage to vascular endothelium and delayed leakage of blood may be the reason for this phenomenon. This is also the common explanation for the occurrence of white-centered hemorrhages, which were detected in 9 of our climbers.[22] These hemorrhages, also known as Roth spots[23] can be found in patients with capillary fragility due to conditions such as systemic infections, sepsis, bacterial endocarditis or anemia. The common underlying etiology seems to be the result of capillary rupture.[22] The white center within the area of bleeding of a Roth spot has been shown histopathologically to represent a fibrin-platelet thrombus. Duane et al. concluded that this white thrombus is at the site of a vessel rupture.[24] Duane et al.[24] and McFadden et al.[8] described the occurrence of Roth spots at high altitudes. In our study mountaineers with Roth spots had significantly lower SpO_2_ values. This supports the possible etiology of retinal bleeding after a hypoxic insult to the retinal vessels at high altitudes, which may cause a wall rupture after a certain time lag.

Climbers with white-centered hemorrhages also had a larger area of retinal bleeding than the others. Mountaineers with only few and small hemorrhages either had no biomicroscopically detectable thrombi or no vessel rupture. This observation may be explained by a distinct pathophysiological process. Reperfusion trauma occurring upon descent to a lower camp may contribute to endothelial damage; induce diathesis and thereafter visible hemorrhages. Hypoxia leads to the production of oxygen free radicals via the activation of neutrophils,[25] such as in ischaemia-reperfusion trauma. These radicals are harmful to the endothelium and increase vascular permeability.[26] Loss of vasomotor control may further contribute to the damage of the retinal vessels.[27] Although these pathophysiological changes were studied in conditions with extensive ischaemia (e.g. surgical procedures), we suggest that due to the exceptionally low oxygen saturation during the climb, this process, even though less pronounced than in total ischaemia, may explain the enhanced occurrence of hemorrhages after descent. The extent of hypoxia during a high altitude climb may determine the morphology of HAR.

Drug intake was shown to have no impact on retinal bleeding in our study. However, only small amounts of different medications were taken by our climbers, which might not have been enough to relevantly influence our analysis.

HAR is most probably the result of systemic hypoxic effects on the eye despite regulatory attempts by the retinal and choroidal circulation. According to our statistical analysis, parameters characterizing ocular circulation, such as retinal and choroidal blood flow and retinal arterial or venous calibers did not play a valid explanatory role for retinal bleeding during a high altitude expedition.

According to our analysis, higher hematocrit, possibly representing higher blood viscosity, correlated positively with the number of hemorrhages. Elevated blood viscosity may increase shear stress to the predamaged retinal vascular endothelial cells.

High altitude retinopathy and its possible etiologies have been described by many authors [28], [29], [30]. A classification for HAR has been established by Wiedman et al.[12]

Although optic disc swelling, which occurred in both groups with ascent to higher altitudes and regressed upon descent, was shown to correlate with total area of hemorrhages during the expedition, the course of these parameters differed at BC2 where almost all optic disc swelling had regressed.[15] Both optic disc swelling and HAR are signs of overall susceptibility to hypoxia. Nevertheless, since reaction of the optic disc to hypoxia is quicker than that of potential retinal hemorrhages, a grading of HAR implying a direct correlation with cerebral attenuation is not warranted. Such a grading of HAR should primarily include optic disc swelling rather than number and area of hemorrhages. Singling out climbers with HACE, no difference in number or area of hemorrhages in comparison to the mountaineers without HACE was detected. This is another indicator that HAR has no prognostic value for cerebral attenuation during a high altitude expedition.

Many reports on high altitude climbing show persistence of symptoms such as scotomas[30] and visual field shrinkage.[31] We found no such sequelae in our climbers 4.5 months after the expedition, nor any signs of visual disturbances during the expedition except for the climber with vitreous hemorrhages in one eye. This may be attributed to the observation that the vast majority of retinal hemorrhages occurred near the optic disc hardly affecting the macula.

Despite the impressive number of ocular changes at high altitudes, it seems that they are mostly transient. We suggest that the number of retinal hemorrhages rather than the total area of bleeding is a delayed marker of overall degree of hypoxic exposure. Also, due to latency of their appearance, retinal hemorrhages should not be considered warning signs of impending severe AMS or HACE. Based on our data, we cannot confirm that the current classification of HAR can be used as a predictor of progressive altitude illness.
